# A Theoretical Approach for Correlating Proteins to Malignant Diseases

**DOI:** 10.3389/fmolb.2020.582593

**Published:** 2020-10-22

**Authors:** Rasha Elnemr, Mohammed M. Nasef, Passant Elkafrawy, Mahmoud Rafea, Amani Tariq Jamal

**Affiliations:** ^1^Climate Change Information Center & Renewable Energy & Expert Systems, Giza, Egypt; ^2^Department of Mathematics and Computer Science, Faculty of Science, Menoufia University, Shibin El Kom, Egypt; ^3^Information Technology and Computer Science, Nile University, Giza, Egypt; ^4^Department of Computer Science, Faculty of Computing and Information Technology, King Abdulaziz University, Jeddah, Saudi Arabia

**Keywords:** malignant tumors, data mining, association rule, *apriori* algorithm, eclat algorithm

## Abstract

Malignant Tumors are developed over several years due to unknown biological factors. These biological factors induce changes in the body and consequently, they lead to Malignant Tumors. Some habits and behaviors initiate these biological factors. In effect, the immune system cannot recognize a Malignant Tumor as foreign tissue. In order to discover a fascinating pattern of these habits, behaviors, and diseases and to make effective decisions, different machine learning techniques should be used. This research attempts to find the association between normal proteins (environmental factors) and diseases that are difficult to diagnose and propose justifications for those diseases. This paper proposes a technique for medical data mining using association rules. The proposed technique overcomes some of the limitations in current association algorithms such as the *Apriori* algorithm and the Equivalence CLAss Transformation (ECLAT) algorithm. A modification to the *Apriori* algorithm has been proposed to mine Erythrocytes Dynamic Antigens Store (EDAS) data in a more efficient and tractable way. The experiments inferred that there is a relation between normal proteins as environment proteins, food proteins, commensal proteins, tissue proteins, and disease proteins. Also, the experiments show that habits and behaviors are associated with certain diseases. The presented tool can be used in clinical laboratories to discover the biological causes of malignant diseases.

## Introduction

Lifestyle habits and behaviors affect human general health, like cigarette smoking, excessive alcohol consumption, excessive sunlight exposure, poor diet, lack of exercise, medical drugs, change of hormones, radiation, viruses, bacteria, and environmental chemicals. Chemical factors might be in the air, water, food, and/or workplace. The genetic makeup is essential so that these mentioned factors can lead to malignant transformation (Fymat, 2017; Iqbal, 2017; Ellberg et al., 2018; Ukawa et al., 2018).

Because of the complicated interplay of many habits and behaviors, it is difficult to predict which combination of these habits and behaviors is accountable for certain cancer. The cause of cancer is still unknown and the human body’s readiness to be diseased is unpredictable. One of the important areas of research today is attempting to identify the association between the habits and behavior of an individual and diseases, specifically, Malignant Tumor.

Rafea and Souchelnytskyi (2012) observed and described phenomena related to the protein content of the Red Blood Cell (RBC). It was noticed that plasma contains antibodies against some of the RBC proteins, which are contained within the cytoplasm of RBC of the same person. The discovery is that RBC has a dynamic store of body antigens [Tissue-Specific Antigens (TSA)], food antigens, environment antigens, bacterial commensals antigens, and disease antigens whether microbial, viral, or tumors. This store is named: Erythrocytes Dynamic Antigens Store (EDAS).

To maximize the utility of the EDAS, computer knowledge processing capability was adopted to increase the profit of this discovery. To this endeavor, a random generation of the EDAS model was described in Rafea et al. (2019). This random generation was based on a mathematical model that simulates reality. The random generation of EDAS consists of a set of normal proteins and a set of disease proteins. The normal proteins are environment proteins, food proteins, commensal proteins, and tissue proteins. The diseases proteins are malignant tumor proteins or pathogens proteins. They developed a biomarker discovery technique to detect a minimum set of biomarkers for each disease. They applied their technique on two categories of diseases; Malignancies (Mi) and Pathogens (Gi). Thus, malignancies have 20 types (M1, M2, …, M20) and pathogens have 20 types (G1, G2, …, G20). In this work, we will use the EDAS data to find which normal proteins (Tissue-Specific Antigens, food antigens, environment antigens, and bacterial commensals antigens) are related to a particular Malignant Tumor. The main challenge of our research is to find the interesting correlations and associations between the set of normal proteins to the set of malignant tumor proteins.

To forecast the association of those biological data, we should use an association rule mining algorithm. The *Apriori* algorithm was described by Agrawal et al. (1993), is widely used to study the relations and associations between items in an ecosystem. The *Apriori* algorithm is simple, and it is easy to program (Ghosh and Dutta, 2016; Patil and Deshmukh, 2016). It applies the *Apriori* property; a candidate itemset is unnecessary if at least one of its subsets is infrequent (Ingle and Suryavanshi, 2015). Hence, it reduces the number of candidate itemsets. However, the *Apriori* algorithm requires multiple scans over the database for generating the itemsets (Ingle and Suryavanshi, 2015; Patil and Deshmukh, 2016). Since the number of database passes is equal to the max length of the frequent itemset, it takes time to scan the database (Han et al., 2000; Mandave et al., 2013; Kaur and Madan, 2015; Rajeswari, 2015). However, the *Apriori* algorithm has low performance in big datasets (Ghosh and Dutta, 2016). In this study, the primary dataset as typical medical data with a considerable number of cases and a large number of features.

Another algorithm is Equivalence CLAss Transformation (ECLAT) was described by Zaki (2000). ECLAT is also used to study the associations between items in a more efficient manner. It only scans the database once. The ECLAT algorithm uses a depth-first search strategy (Shah and Patel, 2015; Shukla and Solanki, 2015; Giri et al., 2016). Thus, it is fast but the accuracy is not preserved, as it violates the *Apriori* property (Shah and Patel, 2015; Ishita and Rathod, 2016). Because the generation of candidate itemset is operated in an equivalence class, the candidate itemsets are not clipped under the prior knowledge. These candidate itemsets still need to be calculated. Although adopting the technology of equivalence classes, ECLAT needs to judge whether two k-itemsets can be joined to generate a (*k* + *1*)-itemsets, a great time is needed if the itemset is very long (Kavitha and Selvi, 2016). A large number of conditional branches are used to merge which are highly predictable. There is a waste of time to calculate the support of infrequent itemsets. It fails to manage the main memory at the time of high candidate itemsets (Giri et al., 2016).

Accordingly, there is a need to propose a technique that preserves the *Apriori* property to ignore a candidate itemset if at least one of its subsets has support less than the threshold and works efficiently to infer association relations. Many researchers have done modifications to improve the efficiency of the *Apriori* algorithm and to overcome some of the limitations of the *Apriori* algorithm. They used some methods to improve *Apriori* efficiency as Intersection (Aqra et al., 2018), Hash-based itemset counting (Park et al., 1995; Vyas and Sherasiya, 2016), Partitioning (Jia et al., 2012), Sampling (Toivonen, 1996; Rajeswari, 2015), etc. The Intersection is a method used to improve memory management, efficiently, by reducing the computation cost of *Apriori* and removing its complexity. The Intersection method is designed for vertical data format, by removing the limitations of the horizontal data format used in *Apriori*. Moreover, the support is calculated by counting the common transactions that contain each element of the candidate set. This takes less time than the original algorithm; however, data must be in a vertical layout (Agrawal et al., 2013; Raval et al., 2013; Aqra et al., 2018).

In this research, we shall enhance the *Apriori* algorithm performance by adopting an intersection mechanism while preserving the *Apriori* property to achieve accuracy. Accordingly, the performance is enhanced by two ways; executing one scan to the database and applying the *Apriori* property that helps to reduce the number of candidate itemsets. Therefore, in this research, the proposed algorithm is given to discover the association of proteins to environmental factors, with higher performance and accuracy.

This paper is structured as follows: Section 2 states the related work. Section 3 states the background of using the *Apriori* algorithm in medical problems. Section 4 includes the proposed model. Section 5 describes the experiment and results. Section 6 describes the evaluation. Section 7 describes the discussion. And Section 8 describes the conclusion.

## Related Work

Some researchers attempt to improve the *Apriori* algorithm based on Intersection as in Ganesh et al. (2016), the authors proposed a Vertical Format Frequent Mining (VFFM) algorithm. This algorithm was used to find frequent items from the database. The transaction database is transformed into a vertical data format. They scan the database only one time. They converted the data into 0 and 1 and calculated the support for them. They used the depth strategy for mining. Thus, they could not apply the *Apriori* property. Also, there is no need for converting the data into 0 and 1 because the researcher did not benefit from it. There is no application for their method.

Aqra et al. (2018) presented an Intermediate Transaction ID *Apriori* (ITD *Apriori*) algorithm where a new itemset format structure is adopted to address the problem of threshold that necessitates rescanning the entire database. This approach creates an intermediate itemset. The intermediate itemset has a new structure called Intermediate Transaction Id itemset ITDM list; this structure increases the efficiency of mining. This can be done by scanning the database and by representing data to a vertical data format. After this process, support can be collected by the intersection TID list. Thus, it improves the overall efficiency as no longer the algorithm needs to rescan the entire database. The algorithm also helps to extract frequent itemsets according to pre-determined minimum support with an independent purpose. Furthermore, the association rule set is extracted with high confidence and weak support. However, they used the depth strategy for mining; thus could not apply the *Apriori* property.

Chen and Xiao (2014) proposed the Intersection Maximum Frequent Pattern (ISMFP) algorithm, which was based on set theory and the idea of a top-down search for mining the maximum frequent patterns. Since the maximum frequent patterns have already implied all frequent patterns. They converted the problem from detecting frequent patterns to discover the maximum frequent patterns, avoiding the production of a large number of candidate sets. They forwarded a kind of association rule mining algorithm depending on the intersection, which decreases the search space and the number of cycles by using the principle of the maximum frequent pattern and intersection. Their experimental results showed that the algorithm ISMFP is efficient in mining frequent patterns; especially there exists a low threshold of support degree or long patterns.

## Background

Multiple papers reviewed the solution of medical problems as Association Rules. They provided a computational study, based on the *Apriori* algorithm to discover the associations among clinical traits and risk factors of different disease [i.e., asthma (Poorani et al., 2018), chronic diseases (Karthiyayini and Jayaprakash, 2015), and heart diseases (Said et al., 2015)]. The *Apriori* algorithm was used to find the frequent symptoms and related causes of a disease from the dataset that was collected from self-reported patients. It was even used in predicting the possibility of chronic occurrence of diseases. In (Karthiyayini and Jayaprakash, 2015), the percentage of possibility for chronic disease was calculated from each symptom of all considered chronic diseases. The higher number of symptoms leads to higher accuracy of calculating the disease possibility.

Others diagnosed by considering NED (No Evidence of Disease) and ED (Evidence of Disease) studies. Fahrudin et al. (2017) experimented with two Association Rules algorithms: *Apriori* and FP-Growth. They categorized NED and ED to detect the relationship between different factors that influences patients. Another study by Gitanjali et al. (2014) was conducted to generate the frequency of diseases that affect patients in various geographical regions and at various periods. The use of association in medicine has numerous critical applications; where mainly *Apriori* is the exemplar algorithm of all those studies.

The state of the art literature concentrated on discovering the relationship between the diseases and their symptoms. However, we have a different goal to discover the relationship between normal proteins and the disease’s proteins.

## Materials and Methods

From the comparison of Association Rule Mining (ARM) algorithms, we propose an enhancement to the *Apriori* algorithm with the concept of vertical format from the ECLAT algorithm. We merged several scientific concepts of mining into *Apriori* employing the set theory of intersection, lattice theory, and vertical data format. Moreover, we use divide and conquer methodology to divide the problem into clusters.

### The Proposed Association Rule Model

The proposed association rule model is used to discover the association between the normal proteins and the diseased proteins. It is composed of two phases. Phase one is the *Disease Sub-Typing (DST)*, we attempt to find the different subtypes of a particular disease. Phase two is the *Association Rule (AR) generation*; we attempt to find the association between each subtype to its normal proteins. Inferring, the association rules of factors causing diseases.

#### Phase One: Disease Sub-Typing

The main idea is to cluster each malignant tumor, M_*i*_, into its subtypes. For each disease, the set of cases is compared to each other to extract similar cases. The similarity is measured by the number of similar proteins. A certain threshold is used to identify the subtype. The rest of the cases are re-evaluated to each other until no further cases remain. The steps are:

1. We select the cases which have the same malignant tumor Mi.
2. For the first case (record), we compare its set of proteins with sets of proteins in the rest cases.
3. The cases which their proteins have similarity with the first case proteins, equal to or greater than the threshold are considered of the same sub-type.
4. The other cases which are less than the threshold are re-evaluated by repeating the previous steps until no further cases remain.

Interestingly, the set of proteins defining a subtype of the disease can be used as a signature of that specific disease for diagnosis.

##### Disease sub-typing algorithm

Algorithm 1: detecting the type of each Disease.


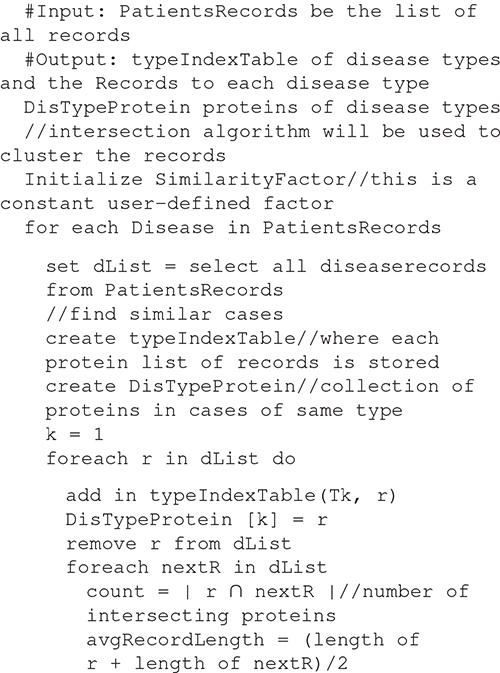



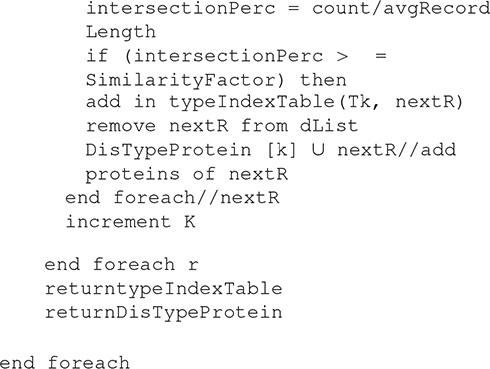


#### Phase Two: Association Rule

The proposed association rule mining algorithm is considered as an enhancement to the *Apriori* algorithm. The proposed algorithm is based on converting the structure of the dataset from horizontal to vertical format. This vertical format will facilitate applying the intersection concept for getting the support for each itemset. Therefore, the breadth-first search strategy of the proposed new algorithm preserves the *Apriori* property. It is known that most association rule mining algorithms that apply the vertical format works by depth search strategy which violates the *Apriori* property. However, our proposed association rule mining algorithm works by the breadth search strategy. From this point, the proposed algorithm does only one scan to the database, decreasing the candidate sets. Thus, it is fast and guarantees accuracy.

### The Proposed Association Rule Mining Algorithm

The main challenge of the model is in its ability to help in finding the relations between the normal proteins and disease conditions that are difficult to diagnose and propose justifications for these diseases through the following steps:

**First**, we scan all the records to convert the datasets from horizontal to vertical format. This step generates a protein index table containing all 1-itemsets (proteins) without repetition, TID (the records where the protein is located), and its support.

**Second**, prune the proteins which don’t satisfy the minimum support.

**Third**, self-join the frequent proteins to generate 2-itemsets. Note that the 2-itemsets are unordered sets such that p1p5 is the same as p5p1. Calculate the support for 2-itemsets by the intersection concept where we get the common records between those 2-itemsets by intersecting the record list of each itemset. This eliminated the scans to the database.

**Forth**, prune the 2-itemsets which don’t satisfy the minimum support.

**Fifth**, self-join the frequent proteins to generate 3-itemsets, however, the ***Apriori* property** has to be applied here. Each subset of the generated 3-itemsets must also be a frequent itemset, i.e. satisfies the minimum support. If at least one 2-itemset of the generated 3-itemsets are infrequent then discard this generated 3-item set.

**Sixth**, prune the 3-itemsets which don’t satisfy the minimum support.

**Finally**, repeat these steps until no new frequent itemsets are identified.

#### Proposed Association Rule Mining Algorithm

Algorithm 2: detecting the association between Normal Proteins and Diseases Proteins.


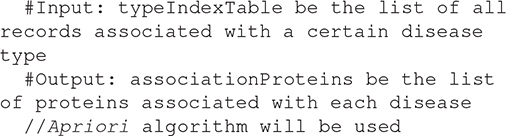



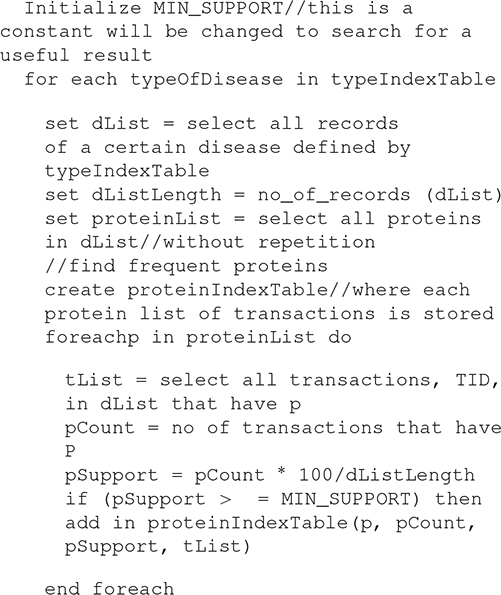



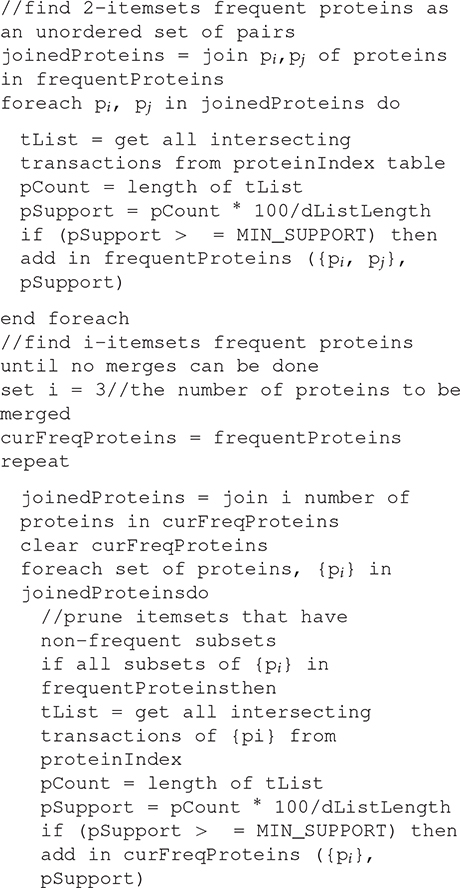



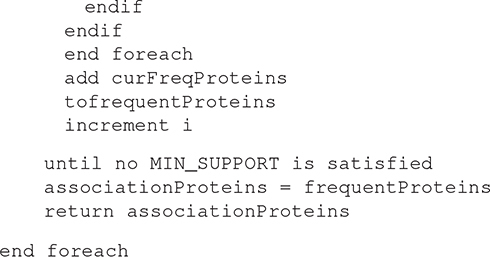


## Experiment and Results

**FIGURE 1:**
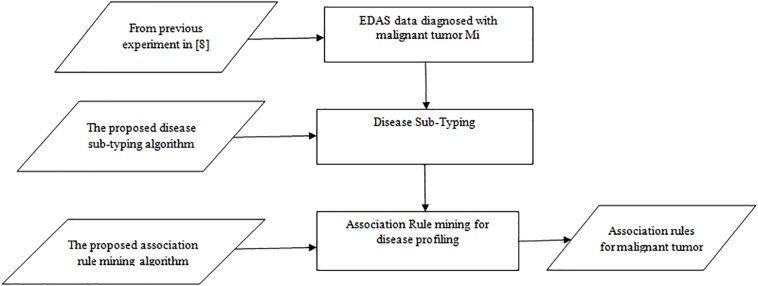
Workflow pipeline of the experiment.

The experiment is divided into three phases as shown in Figure 1: the random generation of the EDAS data, the Disease Sub-Typing phase, and the association rule mining phase. The aim is to detect the association between Normal Proteins and Malignant Tumor Proteins.

The experiment is performed on MacBook Pro, 2.9 GHz Intel Core i5 and 8 GB of RAM, the database is created in Microsoft SQL Server 2008, the algorithms are implemented in C#.

***Phase one***: the generation of EDAS data, our data is based on the previous experiment by Rafea et al. (2019), where the total generated cases are 100K record. Malignant tumor patients are 41,742 records from the total 100 K, which was concluded from the experiment by Rafea et al. (2019). Malignant Tumor (Mi) has 20 types (M1, M2, …, M20). These patients are divided according to their malignant type as shown in Table 1. For example, the number of patients who have a Malignant Tumor (M1) is 2063. The number of patients who have a Malignant Tumor (M7) is 2062. The number of patients who have a Malignant Tumor (M18) is 2080. The number of patients who have a Malignant Tumor (M20) is 2181.

**TABLE 1:** Results of the experiment for malignant tumors (Rafea et al., 2019).

Disease	M1	M2	M3	M4	M5	M6	M7	M8	M9	M10
Number of_records	2063	2109	2083	2053	2035	2094	2062	2135	1982	2096
Disease	M11	M12	M13	M14	M15	M16	M17	M18	M19	M20
Number of_records	2040	2084	2076	2149	2130	2115	2059	2080	2116	2181

***Phase two***: apply the Disease Sub-Typing Algorithm to detect the sub-type of each malignant tumor (Mi) as explained in section 4.1.1

***Phase three:*** The aim is to detect the association between Normal Proteins and Malignant Tumor Proteins for each subtype of a malignant tumor. We shall apply the Association Rule Mining Algorithm to the resulted data from phase 2 as defined in section 4.1.2

### Results

From phase 2, we concluded that each disease can be divided into several subtypes as shown in Table 2. For example,

**TABLE 2:** Results of phase two (malignant tumor subtypes).

Disease	Its subtypes	Number of subtypes
Malignant Tumor (M1)	M1T1, M1T2, M1T3, M1T4, and M1T5	5
Malignant Tumor (M7)	M7T1, M7T2, M7T3, M7T4, M7T5, and M7T6	6
Malignant Tumor (M18)	M18T1, M18T2, M18T3, M18T4, M18T5, and M18T6	6
Malignant Tumor (M20)	M20T1, M20T2, M20T3, M20T4, M20T5, M20T6, and M20T7	7

Malignant Tumor (M1) has 5 subtypes which are M1T1, M1T2, M1T3, M1T4, and M1T5. Malignant Tumor (M7) has 6 subtypes which are M7T1, M7T2, M7T3, M7T4, M7T5, and M7T6.

Malignant Tumor (M18) has 6 subtypes which are M18T1, M18T2, M18T3, M18T4, M18T5, and M18T6.

Malignant Tumor (M20) has 7 subtypes which are M20T1, M20T2, M20T3, M20T4, M20T5, M20T6, and M20T7.

From phase three, we concluded that there are interesting associations between malignant tumor proteins and the environmental factors (normal proteins) as shown in Table 3. For example,

**TABLE 3:** Results of phase three (association rule mining).

Disease	Disease Subtype	Associations between proteins	
		
		Normal proteins	Diseased proteins
Malignant Tumor (M1)	M1T1	P3580	P119913, P119662, P119786, P119535, P119939
Malignant Tumor (M1)	M1T2	P3887	P119640, P119513, P119637, P119515, P119790, P119541, P119917, P119886, P119792, P119668
Malignant Tumor (M7)	M7T5	P3734, P6006	P719501, P719807
Malignant Tumor (M7)	M7T6	P625, P3886	P719785, P719964
Malignant Tumor (M18)	M18T2	P6376	P1819795, P1819919, P1819668, P1819546
Malignant Tumor (M18)	M18T5	P327, P3479	P1819668, P1819696
Malignant Tumor (M20)	M20T3	P777	P2019891, P2019964, P2019765, P2019863, P2019640, P2019643, P2019516, P2019741, P2019614, P2019518, P2019638, P2019889, P2019767, P2019990

(1) In Malignant Tumor (M1) which has a subtype (M1T1) and minimum support%60, there is a relation between the food proteins and the disease proteins as the following (P3580, P119913, P119662, P119786, P119535, P119939), where P3580 is a food protein and the rest are malignant tumor proteins.
(2) In Malignant Tumor (M1) which has subtype (M1T2) and minimum support%60, there is a relation between the food proteins and the disease proteins as the following (P3887, P119640, P119513, P119637, P119515, P119790, P119541, P119917, P119886, P119792, P119668), where P3887 is a food protein and the rest are malignant tumor proteins.
(3) In Malignant Tumor (M7) which has subtype (M7T5) and minimum support%60, there is a relation between the food proteins, the commensal proteins, and the disease proteins as the following (P3734, P6006, P719501, P719807), where P3734 is a food protein, P6006 is a commensal protein and the rest are malignant tumor proteins.
(4) In Malignant Tumor (M7) which has subtype (M7T6) and minimum support%60, there is a relation between the environment proteins, the food proteins, and the disease proteins as the following (P625, P3886, P719785, P719964), where P625 is an environment protein, P3886 is a food protein and the rest are malignant tumor proteins.
(5) In Malignant Tumor (M18) which has a subtype (M18T2) and minimum support%60, there is a relation between the commensal proteins and the disease proteins as the following (P6376, P1819795, P1819919, P1819668, P1819546), where P6376 is a commensal protein and the rest are malignant tumor proteins.
(6) In Malignant Tumor (M18) which has a subtype (M18T5) and minimum support%60, there is a relation between the commensal proteins and the disease proteins as the following (P327, P3479, P1819668, P1819696), where P327 is an environmental protein, P3479 is a food protein and the rest are malignant tumor proteins.
(7) In Malignant Tumor (M20) which has subtype (M20T3) and minimum support%60, there is a relation between the environment proteins, and the disease proteins as the following (P777, P2019891, P2019964, P2019765, P2019863, P2019640, P2019643, P2019516, P2019741, P2019614, P2019518, P2019638, P2019889, P2019767, P2019990), where P777 is an environment protein, and the rest are malignant tumor proteins.

### Rule Generation

The developed algorithm with a lower minimum support threshold allows for more rules to show up. For example, for the disease M18T5, the generated rules are fifteen rules. Consequently, by applying the statistical measure, like confidence, lift, and leverage, the rules are reduced to four main rules. The generated association rules and their confidence, lift, and leverage of M18T5 are shown in Table 4.

**TABLE 4:** Rules, confidence, lift, and leverage.

Rule Number	Rule	Confidence	Lift	Leverage
R1	P3479 ^ P327 → P1819668	86.36%	1.2164	0.0711
R2	P3479 ^ P327 → P1819696	84.81%	1.1751	0.0622
R3	P3479 → P1819696	81.48%	1.1111	0.0533
R4	P347 9→ P1819668	81.48%	1.062	0.0433

Notice that in Table 4, the results of R1 show that disease M18T5 profiled by protein P1819668 is affected by proteins

P3479 and P327 which are food and environmental proteins respectively. R2 shows that disease M18T5 profiled by protein P1819696 is affected by proteins P3479 and P327 which are food and environmental proteins respectively. R3 means that disease M18T5 profiled by protein P1819696 is affected by protein P3479 which is food protein. R4 means that disease M18T5 profiled by protein P1819668 is affected by protein P3479 which is food protein.

## Evaluation

The evaluation between the three algorithms (*Apriori* Algorithm, ITDApriori, Proposed Algorithm) consists of two steps; firstly, the common evaluation methodology by calculating precision, recall, F-measure, and accuracy. Secondly, we experiment the performance by calculating the execution time of the three algorithms (*Apriori* Algorithm, ITDApriori, Proposed Algorithm). Thus, the algorithms have been tested over different minimum support and different number of transactions (cases). The minimum support values used are 40%, 50%, 60, and 70%, for the number of transactions 500, 1000, and 1500. For example, we will take (M1T2) to explain the results.

Table 5 shows the results of the first step of the evaluation methodology on several experiments covering different minimum support (40%, 50%, 60%, and 70%) on the *Apriori* Algorithm, the ITDApriori Algorithm, and the proposed ARM algorithm. As shown in Table 5, we noticed that the precision, recall, f-measure, and accuracy are the same for the three algorithms. This is due to the three algorithms gave the same frequent itemset. By which accuracy is not violated by our proposed algorithm.

**TABLE 5:** Precision, recall, f-measure, and accuracy of the three algorithms on different support (40%, 50%, 60%, and 70%).

Algorithm	Precision %				Recall %				F-measure %				Accuracy %			
				
	40%	50%	60%	70%	40%	50%	60%	70%	40%	50%	60%	70%	40%	50%	60%	70%
*Apriori* Algorithm	42	54	62	64	67	69	84	87	52	61	71	74	91	95	98	98
ITDApriori	42	54	62	64	67	69	84	87	52	61	71	74	91	95	98	98
Proposed Algorithm	**42**	**54**	**62**	**64**	**67**	**69**	**84**	**87**	**52**	**61**	**71**	**74**	**91**	**95**	**98**	**98**

**For the second step of the evaluation**, we evaluated the performance of time between the three algorithms. We applied three experiments that are done based on changing the number of transactions and the minimum support values.

**FIGURE 2:**
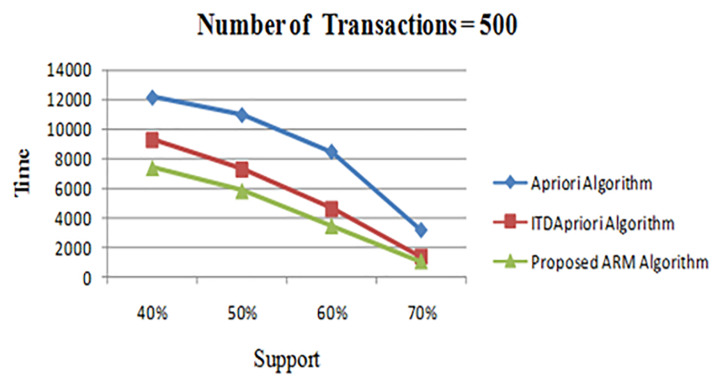
Execution times over different support: *Apriori* Algorithm ITD *Apriori* proposed Algorithm on 500 transactions.

The first experiment is carried out for the three algorithms (*Apriori* Algorithm, ITDApriori, and the Proposed Algorithm) on 500 transactions. From Table 6, we found that whatever the minimum support value the proposed ARM algorithm is the best. Table 6 is visualized in Figure 2.

**TABLE 6:** The execution time (in second) comparison among *Apriori* Algorithm, ITDApriori, Proposed ARM Algorithm on 500 transactions.

Minimum Support	*Apriori* (in sec.)	ITDApriori (in sec.)	Proposed ARM (in sec.)	Improvement %
40%	12174	9296	**7407**	**44.93**
50%	10979	7312	**5840**	**56.60**
60%	8472	4620	**3448**	**89.85**
70%	3125	1368	**1035**	**117.05**

**FIGURE 3:**
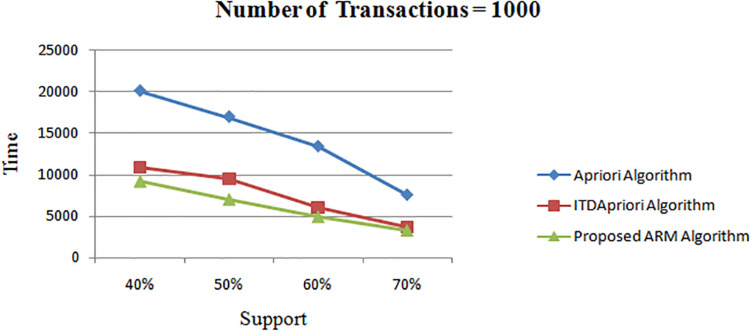
Execution times over different support: *Apriori* Algorithm ITD *Apriori* proposed Algorithm on 1000 transactions.

The second experiment is conducted for the three algorithms over 1000 transactions. The execution time for the three algorithms is presented in Table 7. We noticed that, although changing the value of minimum support, the proposed ARM algorithm is the best. Table 7 is represented as a graph in Figure 3.

**TABLE 7:** The execution time (in second) comparison among *Apriori* Algorithm, ITDApriori, Proposed ARM Algorithm on 1000 transactions.

Minimum Support	*Apriori* (in sec.)	ITDApriori (in sec.)	Proposed ARM (in sec.)	Improvement (%)
40%	20067	10849	**9195**	**68.11**
50%	16955	9452	**7015**	**88.22**
60%	13433	6028	**4910**	**98.18**
70%	7628	3730	**3275**	**118.71**

**FIGURE 4:**
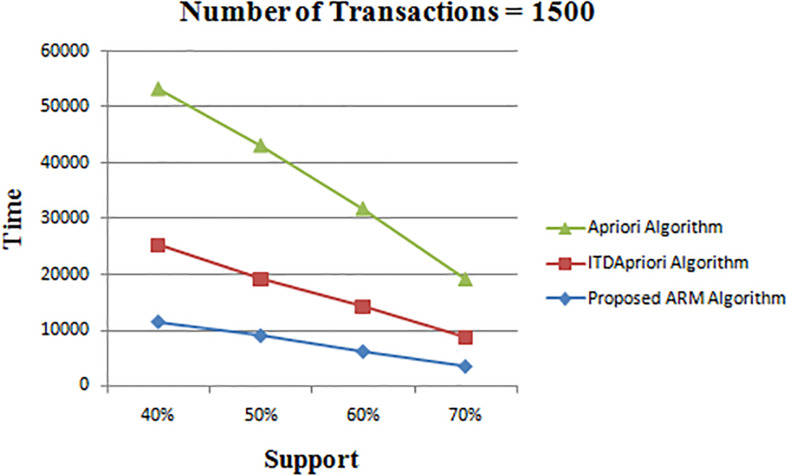
Execution times over different support: *Apriori* Algorithm ITD *Apriori* proposed Algorithm on 1500 transactions.

Thirdly, the experiment is done for the three algorithms over 1500 transactions. The execution time is presented in Table 8. We found that whatever the minimum support value the proposed ARM algorithm is the best. Table 8 is depicted as a graph in Figure 4.

**TABLE 8:** The execution time (in second) comparison among *Apriori* Algorithm, ITDApriori, Proposed ARM Algorithm on 1500 transactions.

Minimum Support	*Apriori* (in sec.)	ITDApriori (in sec.)	Proposed ARM (in sec.)	Improvement %
40%	28176	13880	**11376**	**84.85**
50%	24013	10277	**8949**	**91.58**
60%	17642	8095	**6134**	**109.79**
70%	10384	5260	**3498**	**123.61**

## Discussion

From the previous experiments, we concluded that the proposed ARM algorithm is efficient than the ITDApriori and the *Apriori* algorithm. When the number of transactions is increased, the proposed ARM algorithm showed high efficiency, and this is evident from the time of implementation. The proposed ARM algorithm converts the data structure from horizontal to vertical data format. It executes one scan to the database to create an index table.

The proposed ARM algorithm does not do a scan over the entire database for calculating the support of candidate itemsets, it scans only the records of the candidate itemsets in the index table, which causes to reduce the search time. The computation of the support is done by getting the intersection of the Transaction Id sets of the corresponding k-itemsets. The proposed ARM algorithm applies the *Apriori* property which plays a vital role in reducing the search time and space when handling properly. By decreasing the number of candidates itemsets, our algorithm does not waste time for calculating the support for infrequent itemsets. Moreover, it uses the breadth-first search strategy on vertical data layout which guarantees accuracy.

The horizontal format of the data in the *Apriori* algorithm leads to some problems: it does several scans to the entire database which is the main cause of increasing the time. Also, when the number of transactions increases the runtime increases. The *Apriori* algorithm is not efficient in case the number of transactions increases and the number of items increases because more candidate itemsets must be examined during candidate generation and support calculation.

Although the ITDApriori algorithm converts the data structure from horizontal to vertical data format and calculating the support of candidate items by intersection, it does not apply the *Apriori* property which leads to an increase in the number of candidate itemsets. Thus, it takes extra time to calculate the support for infrequent items. Moreover, the larger the number of items the higher the storage space required.

The *Apriori* algorithm scans the database too many times, which reduces the overall performance. Due to this, both the time and space complexity of the *Apriori* algorithm are very high: O (2^*D*^), thus exponential, where D is the width of the transaction (the total number of items) present in the database. While in our proposed Association Rule Mining algorithm the complexity is O (D + D^2^). In conclusion, we can say that the proposed ARM Algorithm is faster than the other algorithms while preserving accuracy.

## Conclusion

This paper is focused on issues related to the design and implementation of an advanced technique. Its main purpose is to help in finding the association between the habits and behavior of the human and causing malignant tumor. The proposed model in this stage is based on hypothetical generated data. Our model is tested by generating databases each with 41742 patients’ records who suffering from malignancies. Firstly, the proposed model detects the sub-type of each disease. Secondly, it finds the relation between the normal proteins and each sub-type of malignancies. Lastly, it presents the evaluation of our proposed ARM algorithm against the original *Apriori* Algorithm and, the Intermediate TID *Apriori* (ITDApriori) Algorithm. The evaluation is based on accuracy and time. The results demonstrate that the proposed algorithm achieves superior performance in execution time with preserving accuracy. In the future, the proposed model will be modified by using a parallel method to take in an extremely large database. Improve the proposed model to work on patients that may be infected by more than one disease. Also, it will more interesting to improve the proposed model to predict the relations between these diseases.

## Data Availability Statement

The raw data supporting the conclusions of this article will be made available by the authors, without undue reservation.

## Author Contributions

RE is a researcher working on disease diagnosis and this work is a part of her Ph.D. thesis. All the authors worked on reviewing the research.

## Conflict of Interest

The authors declare that the research was conducted in the absence of any commercial or financial relationships that could be construed as a potential conflict of interest.

## References

[B1] Agrawal P. Kashyap S. Pandey V. C. Keshri S. P. (2013). A review approach on various form of apriori with association rule mining. *Int. J. Recent Innov. Trends Comput. Commun.* 1 462–468.

[B2] Agrawal R. Imielinski T. Swami A. N. (1993). Mining association rules between sets of items in large databases. *ACM SIGMOD Rec.* 22 207–216. 10.1145/170036.170072

[B3] Aqra I. Herawan T. Ghani N. A. Akhunzada A. Ali A. Razali R. B. (2018). A novel association rule mining approach using TID intermediate itemset. *PLoS One* 13:e0179703. 10.1371/journal.pone.0179703 29351287 PMC5774682

[B4] Chen X. Xiao J. (2014). Association rules algorithm based on the intersection. *Open Cybernet. Syst. J.* 8 1152–1157. 10.2174/1874110X01408011152

[B5] Ellberg C. Olsson H. Jernström H. (2018). Current smoking is associated with a larger waist circumference and a more androgenic profile in young healthy women from high-risk breast cancer families. *Cancer Causes Control* 29 243–251. 10.1007/s10552-017-0999-3 29299723 PMC5794810

[B6] Fahrudin T. M. Syarif I. Barakbah A. R. (2017). “Discovering patterns of NED-breast cancer based on association rules using apriori and FP-growth,” in *Proceedings of the International Electronics Symposium on Knowledge Creation and Intelligent Computing (IES-KCIC)*, Bali, 132–139.

[B7] Fymat A. L. (2017). Genetics, epigenetics and cancer. *Cancer Ther. Oncol. Int J.* 4:555634.

[B8] Ganesh C. Sathyabhama B. Geetha D. T. (2016). Fast frequent pattern mining using vertical data format for knowledge discovery. *Int. J. Eng. Res. Manag. Technol.* 5 141–149.

[B9] Ghosh A. Dutta A. (2016). Comparative study of different improvements of apriori algorithm. *Int. J. Recent Innov. Trends Comput. Commun.* 4 75–78. 10.17762/ijritcc.v4i3.1837

[B10] Giri R. Bhatt A. Bhatt A. (2016). Frequent pattern mining algorithms analysis. *Int. J. Comput. Appl.* 145 33–36. 10.5120/IJCA2016910763 7911558

[B11] Gitanjali J. Ranichandra C. Pounambal M. (2014). Apriori algorithm based medical data mining for frequent disease identification. *IPASJ Int. J. Inform. Technol.* 2 1–5.

[B12] Han J. Pei J. Yin Y. (2000). Mining frequent patterns without candidate generation. *ACM SIGMOD Rec.* 29 1–12. 10.1145/335191.335372

[B13] Ingle M. G. Suryavanshi N. (2015). Association rule mining using improved apriori algorithm. *Int. J. Comput. Appl.* 112 37–42.

[B14] Iqbal A. (2017). Effect of food on causation and prevention of gastric cancer. *J. Cancer Prev. Curr. Res.* 8:00289 10.15406/jcpcr.2017.08.00289

[B15] Ishita R. Rathod A. (2016). ECLAT with large database parallel algorithm and improve its efficiency. *Int. J. Comput. Appl.* 143 33–37. 10.5120/ijca2016910462 7911558

[B16] Jia Y. Xia G. Fan H. Zhang Q. Li X. (2012). “An improved apriori algorithm based on association analysis,” in *Proceedings of the 3rd IEEE International Conference (ICNDC)*, Washington, DC, 208–211.

[B17] Karthiyayini R. Jayaprakash J. (2015). Association technique on prediction of chronic diseases using apriori algorithm. *Int. J. Innov. Res. Sci. Eng. Technol.* 4 255–259.

[B18] Kaur J. Madan N. (2015). Review of Apriori Algorithm and its Recent Improvements. *Int. J. Emerg. Technol. Comput. Appl. Sci.* 12 150–152.

[B19] Kavitha M. Selvi S. T. (2016). Comparative study on apriori algorithm and Fp growth algorithm with pros and cons. *Int. J. Comput. Sci. Trends Technol.* 4 161–164.

[B20] Mandave P. Mane M. Patil S. (2013). Data mining using Association rule based on APRIORI algorithm and improved approach with illustration. *Int. J. Latest Trends Eng. Technol.* 3 107–113.

[B21] Park J. S. Chen M. S. Yu P. S. (1995). An effective hash-based algorithm for mining association rules. *ACM SIGMOD Rec.* 24 175–186. 10.1145/568271.223813

[B22] Patil S. D. Deshmukh R. (2016). Review and analysis of apriori algorithm for association rule mining. *Int. J. Latest Trends Eng. Technol.* 6 104–112.

[B23] Poorani S. Balasubramanie P. Kumar D. V. (2018). Apriori algorithm for identifying the association rules between clinical traits of asthma. *Int. J. Pure Appl. Math.* 118 4695–4706.

[B24] Rafea M. ELkafrawy P. Nasef M. Elnemr R. Jamal A. T. (2019). Applying machine learning of erythrocytes dynamic antigens store in medicine. *Front. Mol. Biosci.* 6:19. 10.3389/fmolb.2019.00019 31001536 PMC6456707

[B25] Rafea M. Souchelnytskyi S. (2012). *Rediscovering Red Blood Cells: Revealing Their Dynamic Antigens Store and Its Role in Health and Disease, in Blood Cell-an Overview of Studies in Hematology. MoschandreouTE.* London: IntechOpen.

[B26] Rajeswari K. (2015). Improved apriori algorithm–a comparative study using different objective measures. *Int. J. Comput. Sci. Inform. Technol.* 6 3185–3191.

[B27] Raval M. R. Rajput I. J. Gupta V. (2013). Survey on several improved Apriori algorithms. *IOSR J. Comput. Eng.* 9 57–61. 10.9790/0661-0945761

[B28] Said I. Haruna A. Garko A. (2015). Association rule mining on medical data to predict heart disease. *Int. J. Sci. Technol. Manag.* 4 26–35.

[B29] Shah A. Patel P. (2015). A Collaborative approach of frequent item set mining: a survey. *Int. J. Comput. Appl.* 107 34–36. 10.5120/18775-0088 7911558

[B30] Shukla R. Solanki A. K. (2015). Performance evaluation for frequent pattern mining algorithm. *Int. J. Eng. Res. Gen. Sci.* 3 910–915.

[B31] Toivonen H. (1996). “September. Sampling large databases for association rules,” in *VLDB ’96: Proceedings of the 22th International Conference on Very Large Data Bases*, Hong Kong, 134–145.

[B32] Ukawa S. Tamakoshi A. Mori M. Ikehara S. Shirakawa T. Yatsuya H. (2018). Association between average daily television viewing time and the incidence of ovarian cancer: findings from the Japan Collaborative Cohort Study. *Cancer Causes Control* 29 213–219. 10.1007/s10552-018-1001-8 29340890

[B33] Vyas K. Sherasiya S. (2016). Modified apriori algorithm using hash based technique. *Int. J. Adv. Res. Innov. Ideas Educ.* 2 1229–1234.

[B34] Zaki M. J. (2000). Scalable algorithms for association mining. *IEEE Trans. Knowl. Data Eng.* 12 372–390. 10.1109/69.846291

